# Translational Initiation at a Non-AUG Start Codon for Human and Mouse Negative Elongation Factor-B

**DOI:** 10.1371/journal.pone.0127422

**Published:** 2015-05-26

**Authors:** Haihui Pan, Xiayan Zhao, Xiaowen Zhang, Mohamed Abouelsoud, Jianlong Sun, Craig April, Asma Amleh, Jian-Bing Fan, Yanfen Hu, Rong Li

**Affiliations:** 1 Department of Molecular Medicine, University of Texas Health Science Center at San Antonio, San Antonio, TX, 78229, United States of America; 2 Department of Biology, School of Sciences and Engineering, The American University in Cairo, New Cairo, 11835, Egypt; 3 Illumina, Inc., San Diego, CA, 92121, United States of America; University of British Columbia, CANADA

## Abstract

Negative elongation factor (NELF), a four-subunit protein complex in metazoan, plays an important role in regulating promoter-proximal pausing of RNA polymerase II (RNAPII). Genetic studies demonstrate that the B subunit of mouse NELF (NELF-B) is critical for embryonic development and homeostasis in adult tissue. We report here that both human and mouse NELF-B proteins are translated from a non-AUG codon upstream of the annotated AUG. This non-AUG codon sequence is conserved in mammalian NELF-B but not NELF-B orthologs of lower metazoan. The full-length and a truncated NELF-B that starts at the first AUG codon both interact with the other three NELF subunits. Furthermore, these two forms of NELF-B have a similar impact on the transcriptomics and proliferation of mouse embryonic fibroblasts. These results strongly suggest that additional amino acid sequence upstream of the annotated AUG is dispensable for the essential NELF function in supporting cell growth *in vitro*. The majority of mouse adult tissues surveyed express the full-length NELF-B protein, and some contain a truncated NELF-B protein with the same apparent size as the AUG-initiated version. This result raises the distinct possibility that translational initiation of mouse NELF-B is regulated in a tissue-dependent manner.

## Introduction

For a large number of RNA polymerase II (RNAPII)-dependent transcriptionally active genes in multicellular organisms, the polymerase is preferentially accumulated in the promoter-proximal region [1–5]. Emerging evidence suggests that pervasive RNAPII pausing is particularly important for timely and synchronous transcriptional activation of genes involved in stress response and tissue development [6–11].

The negative elongation factor (NELF) is a critical regulator of RNAPII pausing [12–19]. NELF is composed of four subunits, A, B, C/D, and E, which are only present in metazoan including *Drosophila* and mammals [13]. The RNAPII-pausing activity of NELF depends on all four NELF subunits, as elimination of any single NELF subunit leads to the loss of NELF function [1,13,20–25].

While the vast majority of published studies of NELF function focus on its RNAPII-pausing activity in cell-based systems *in vitro*, recent work in genetic models *in vivo* provides definitive evidence for an important role of NELF during embryogenesis in both mouse [26,27] and *Drosophila* [28,29]. Furthermore, using an inducible knockout mouse model, we recently showed that NELF-B in adult mice was required for energy metabolism-related transcription and normal cardiac function [30]. Thus, it is likely that NELF-dependent RNAPII pausing is involved in supporting tissue development and homeostasis in response to diverse environmental and physiological stimuli.

In the current study, we report that translation of human NELF-B and its mouse ortholog initiates from a non-AUG codon upstream of the annotated AUG. We further showed that both full-length NELF-B and the truncated, AUG-initiated protein support cell proliferation *in vitro* and share similar transcriptomics in mouse embryonic fibroblasts (MEF). Most adult mouse tissues surveyed express the full-length NELF-B protein. Our study points to possible regulation of alternative translational initiation of NELF-B *in vivo*.

## Results and Discussion

### The annotated AUG initiation codon of mouse Nelf-b is dispensable for translation

**Fig 1:**
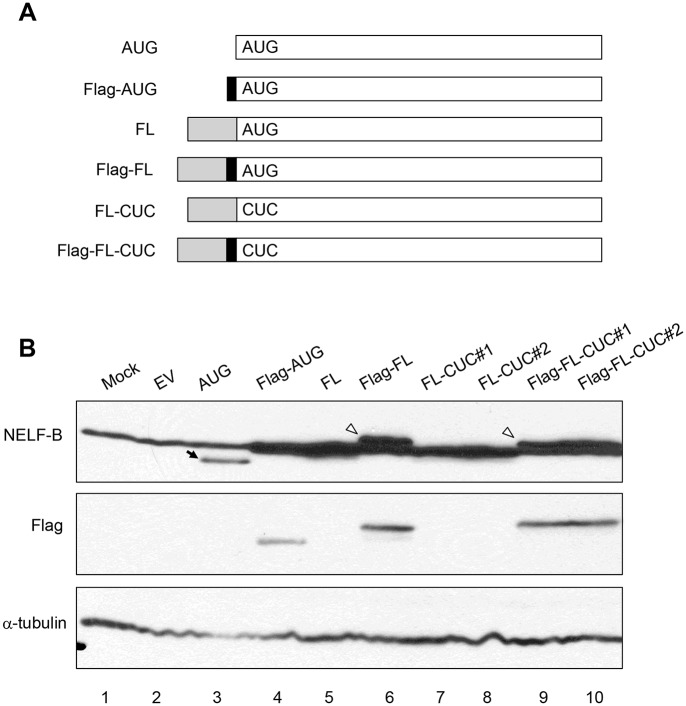
The first AUG codon in mouse *Nelf-b* gene is dispensable for full-length protein translation. (**A**) Diagram showing various NELF-B expression constructs. “AUG” contains the NELF-B coding sequence starting at the annotated initiating AUG. The solid box denotes the location of the Flag peptide. “FL” contains the entire 5’-UTR sequence of *Nelf-b*. The “CUC” construct has the mutation in the annotated AUG codon. (**B**) NELF-B and Flag western blot showing the expression of the constructs described in (A) in 293T cells. Two independent clones of “FL-CUC” and “Flag-FL-CUC” were included.

When expressing a mouse *Nelf-b* cDNA clone that started at the first AUG annotated by the National Center for Biotechnology Information (NCBI) as the translation initiation codon (Fig 1), we noticed that the resulting untagged protein migrated faster than the endogenous NELF-B protein in MEF (Fig 1). We then included in the cDNA clone the entire exon 1, which contains the annotated AUG and 5’untranslated region (5’UTR). The ectopically expressed protein from the longer cDNA clone co-migrated with endogenous NELF-B (Fig 1). The size difference between the FL and AUG versions was confirmed when both ectopic versions were tagged with the Flag epitope and detected by the anti-Flag antibody (Fig 1). This suggests that translation of the full-length (FL) NELF-B protein is initiated upstream of the annotated AUG. In further support, mutation of the annotated AUG to CUC did not affect the product of the full-length (FL) cDNA, either the untagged (lanes 7 and 8) or Flag-tagged version (lanes 9 and 10) (Fig 1).

### A conserved non-canonical CUC codon is important for translation of full-length NELF-B

**Fig 2:**
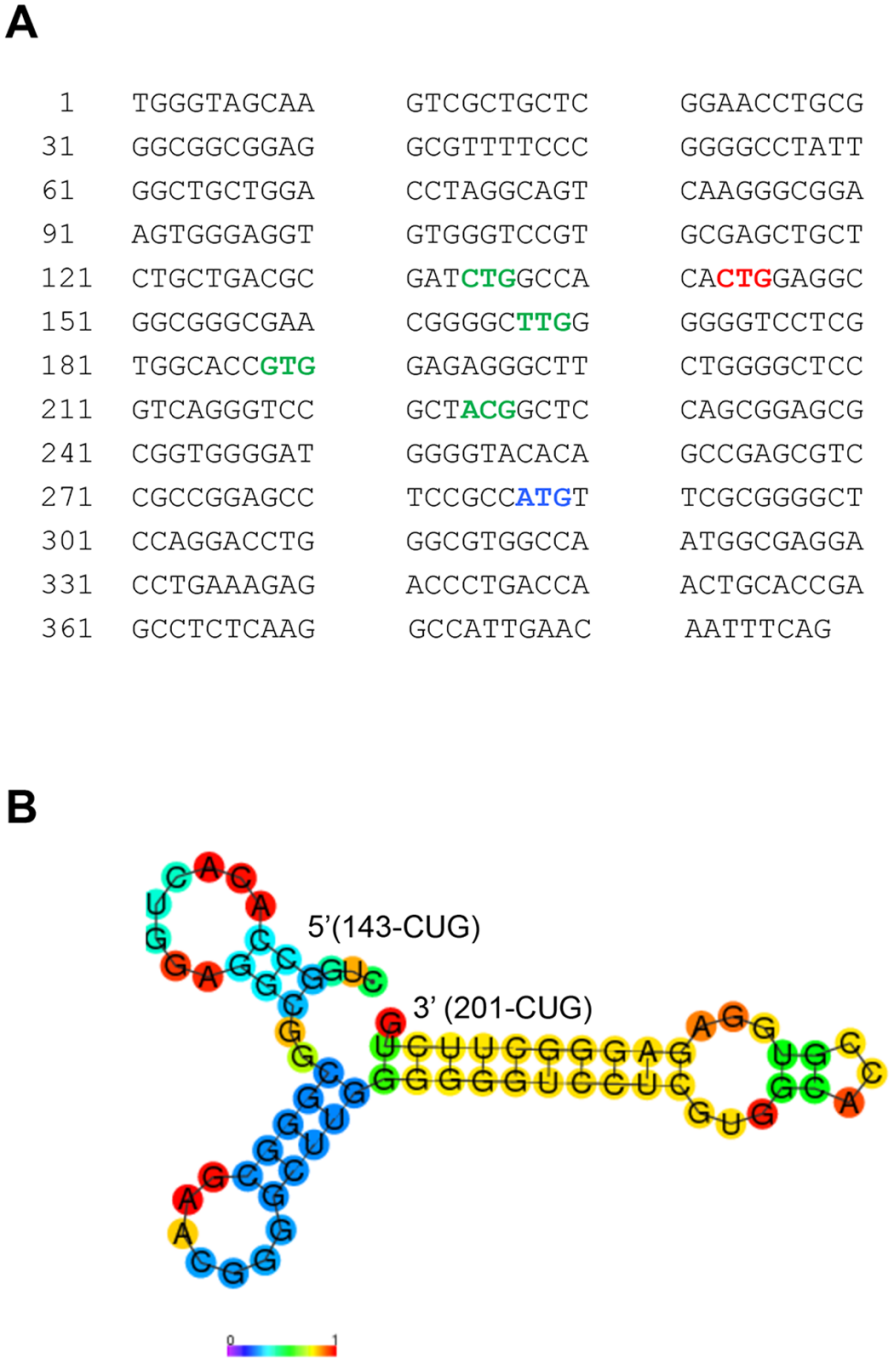
Sequence of the first exon of mouse *Nelf-b* gene. (**A**) Codons of interest are highlighted in red (CUG-143), green (other potential translational initiation alternative codons), and blue (annotated initiating AUG codon). (**B**) RNA folding and hybridization prediction for the 70 bp sequence downstream of CUG-143 in the 5’-UTR of mouse *Nelf-b* (ΔG = -24.20 Kcal/mol).

The optimal context for AUG codon in mammals is GCCRCCAUGG (the Kozak motif), in which the purine at -3 position (R; but adenine preferred) and guanidine (G) at +1 position (relative to “A” of the AUG) are the most important surrounding residues [31,32]. Published work has shown that certain eukaryotic proteins use non-AUG as the initiation codon [33]. Non-AUG codons, with CUG being the most efficient in mammals, appear to have the same preference for the optimal context as does the AUG codon [34–37]. Several of these previously reported non-AUG initiation codons are indeed present in exon 1 of *Nelf-b*, upstream of the annotated first AUG and in-frame with the NELF-B open reading frame, (Fig 2). Among these potential non-AUG codons, CUG-143 (in red) has the surrounding sequence (GCCACACUGG) most closely matching the consensus Kozak motif and is therefore expected to be the most efficient translation initiation site. In comparison, the flanking sequence of the annotated AUG, UCCGCCAUGU, provides less favorable context for initiation. Furthermore, previous work indicates that a strong RNA secondary structure downstream of a non-AUG initiation codon can substantially enhance translational efficiency [38]. In support, RNA folding and hybridization software [39] predicts a favorable secondary structure formed by the 5’-UTR sequences downstream of the CUG-143 in both mouse *Nelf-b* and its human ortholog (Fig 2 and data not shown).

**Fig 3:**
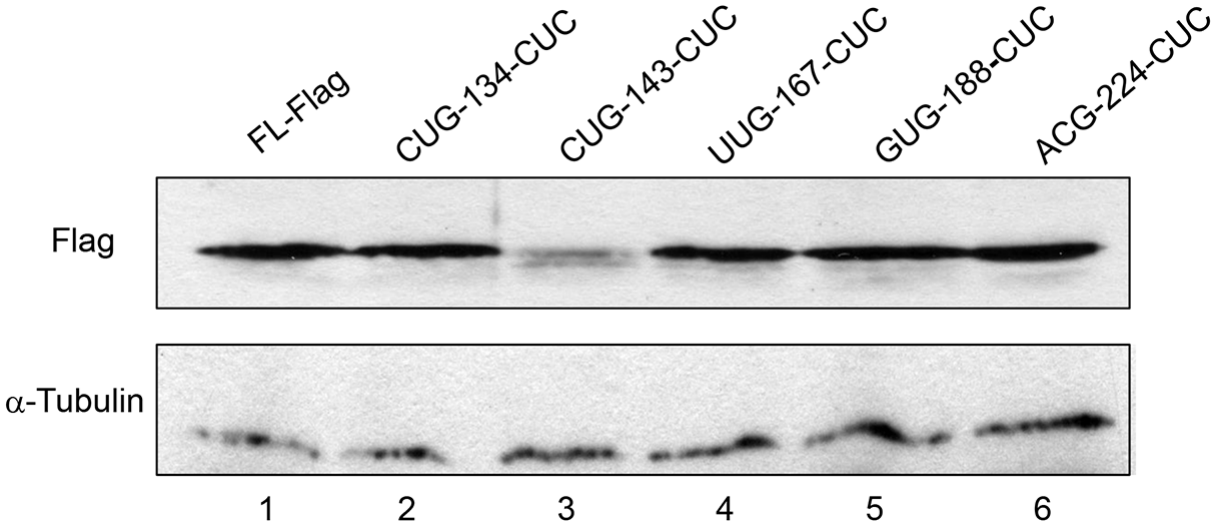
A non-canonical codon is important for translation of full-length NELF-B. Expression of NELF-B protein with various point mutations at different alternative initiation codons in MEFs.

To experimentally validate the importance of CUG-143 in NELF-B translation, we used site-directed mutagenesis to change CUG to CUC. The point mutation significantly reduced intensity of full-length NELF-B, without significantly boosting the usage of the downstream canonical AUG codon (lane 3) (Fig 3). In contrast, mutations of the other putative non-AUG codons did not affect the expression of the full-length protein (Fig 3). Utilization of CUG-143 as the initiation codon for NELF-B is predicted to yield a polypeptide of 628 amino acids (aa), 48 aa longer than the AUG-initiated one. There are at least 42 genes in mammals that are reported to have non-AUG initiation codons [40]. Most of these cases contain a non-AUG codon in addition to the canonical AUG codon, thus resulting in both short and long protein isoforms from the same transcript via a “leaky scanning” mechanism [41]. In contrast, when ectopically expressed in MEF, the canonical AUG codon of mouse *Nelf-b* was not efficiently utilized when the upstream non-AUG codon was mutated. However, the CUG-143-CUC mutant construct still gave rise to a weak band at the FL position, which could be due to inefficient utilization of the mutated CUC codon or a nearby alternative non-AUG codon.

**Fig 4:**
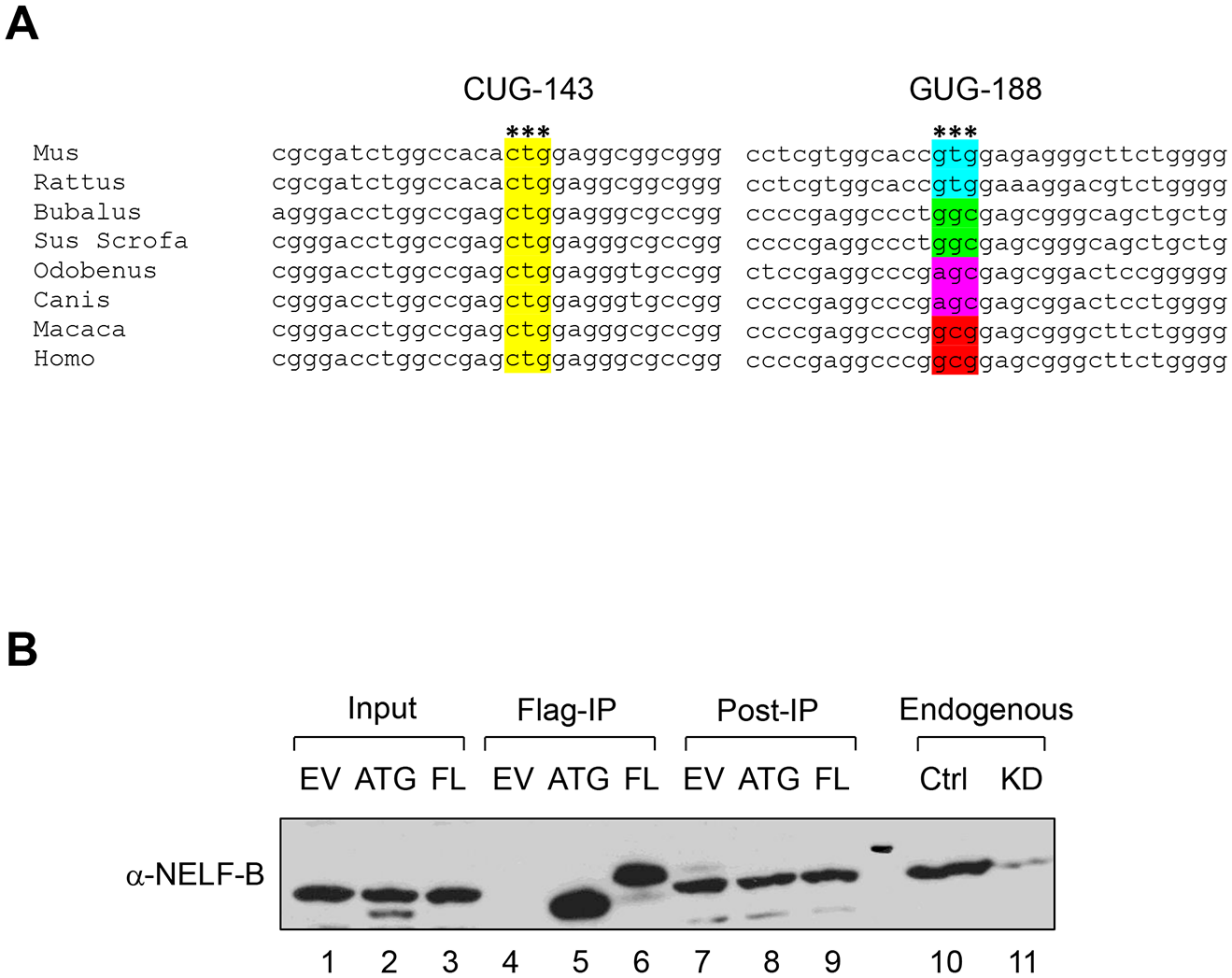
The non-AUG initiation codon is conserved among mammals. (**A**) Sequence alignment around the CUG-143 and GUG-188 codons. (**B**) Western blot showing that 5’-UTR of Human NELF-B is required for full-length protein translation.

The mRNA transcripts of all mammalian *Nelf-b* have at least 200 bp upstream of the AUG codon, in contrast to 78 bp in flies. In particular, the nucleotide sequence surrounding CUG-143 is highly conserved among mammals (Fig 4). Like mouse *Nelf-b*, a cDNA clone of human *NELF-B* containing the entire 5’UTR produced a polypeptide that migrated more slowly than the one initiated from the corresponding annotated AUG (Fig 4). Thus human full-length NELF-B protein is likely translated from an upstream non-AUG codon in a similar fashion as its mouse counterpart.

Recent global mapping of translational initiation sites in mammalian cells revealed previously unannotated, potential alternative translation initiation sites at the non-AUG codons [33,42]. Interestingly, both published studies showed that CUG was the most frequently used among all the non-AUG codons detected. Of note, the whole-proteome study of mouse embryonic stem cells (mESC) by Ingolia *et al*. reported one in-frame non-canonical translation initiation site for mouse *Nelf-b*, GUG-188, which is 33 codons upstream of the annotated AUG (Fig 2) [33]. However, mutation of this codon in our study did not affect translation of the FL protein in MEF (Fig 3). The discrepancy could be due to the different cell types used in the two studies (mESC vs. MEF). However, because GUG-188 is not conserved between mice and humans (Fig 4), it is more likely that CUG-143 as defined in our study represents the authentic non-AUG initiation codon.

### Functional comparison between the FL and AUG versions of mouse NELF-B

**Fig 5:**
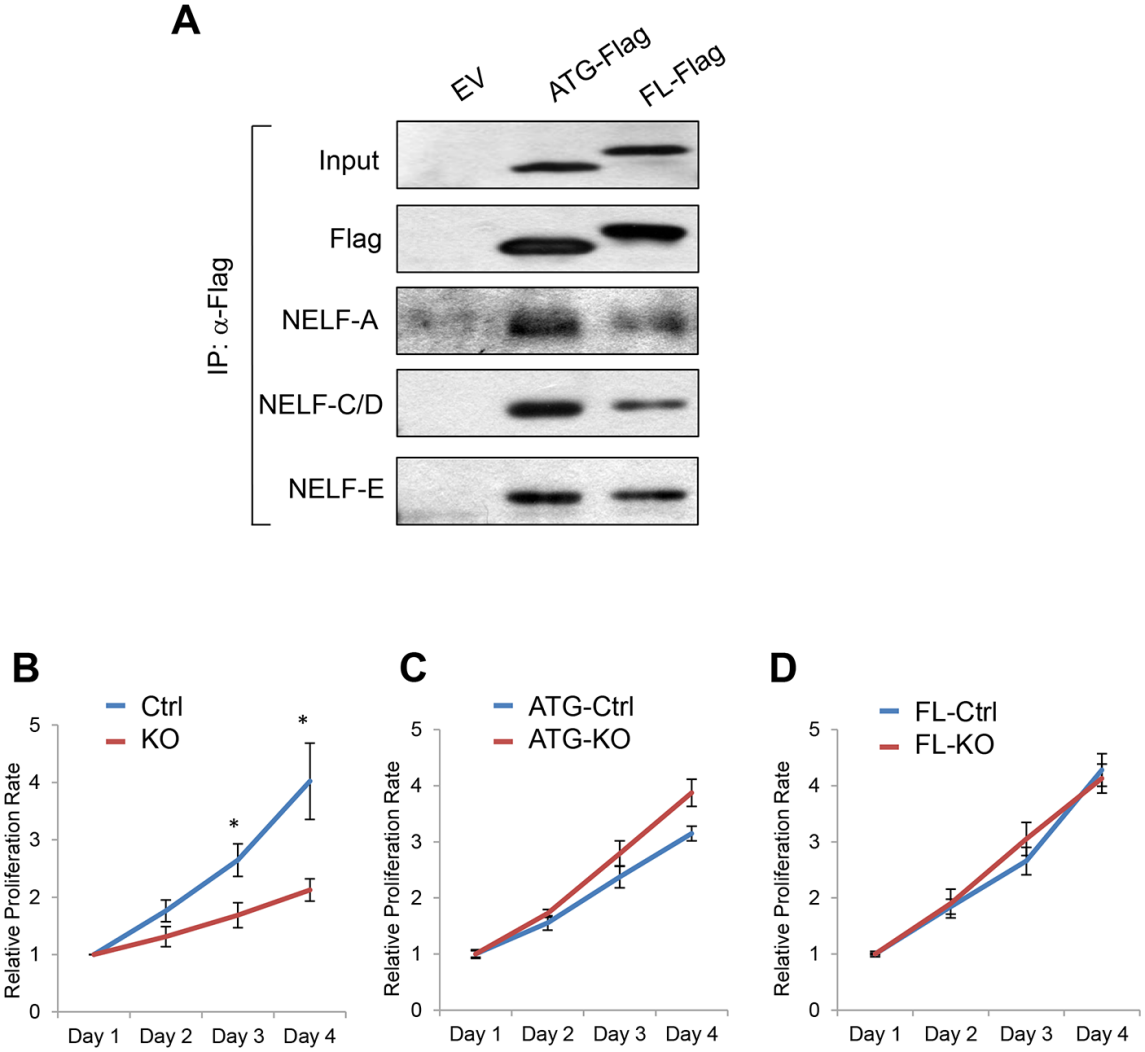
Both full-length and AUG-initiated NELF-B are capable of supporting proliferation of mouse embryonic fibroblasts. (**A**) Western blots indicating both AUG and FL NELF-B were capable of interacting with the other NELF subunits in MEFs. (**B**) Growth curve for WT and *Nelf-b* KO MEFs. (**C**) Growth curve showing AUG-NELF-B expressing cells in the WT and KO background. (**D**) Growth curve showing FL-NELF-B expressing cells in the WT and KO background. * p < 0.05.

Usage of non-canonical initiation codons is much more common in lower organisms such as viruses and bacteria than in mammals. Given that NELF-B is only present in metazoan and that the 5’UTR sequence is not conserved between flies and mammals, it is tempting to speculate that utilization of the non-AUG codon in NELF-B could confer functionality specific for mammalian development and homeostasis. To explore any functional difference between the FL and AUG versions of NELF-B, we first compared their association with the other three NELF subunits. Co-immunoprecipitation (co-IP) showed that the AUG version of NELF-B retained the affinity of the FL version for NELF-A, C/D, and E (Fig 5). We previously demonstrated that *Nelf-b* ablation in MEF significantly compromised cell proliferation [43] (Fig 5). By ectopically expressing either the FL or AUG version of NELF-B, we were able to rescue the proliferative defect of the *Nelf-b* knockout (KO) MEF (Fig 5), suggesting that the extra 48 aa in FL NELF-B is dispensable for NELF-B dependent cell growth *in vitro*. Consistent with the proliferation assay, the number of differentially expressed genes between FL and AUG-expressing *Nelf-b* KO cells (193) is far fewer than what was reported previously (1,444) between wild-type (WT) and *Nelf-b* KO MEF [43] (S1 Table). Over 95% of genes affected in *Nelf-b* KO were unchanged between the FL and AUG-NELF-B expressing MEF. Furthermore, gene ontology analysis indicates that cell growth/death-related genes, which are enriched in *Nelf-b* WT/KO expression profiling [43], are not over-represented in the FL/AUG gene set. Based on these data, we conclude that initiation from a non-AUG codon does not provide additional sequence important for NELF-B to support cell growth *in vitro*.

**Fig 6:**
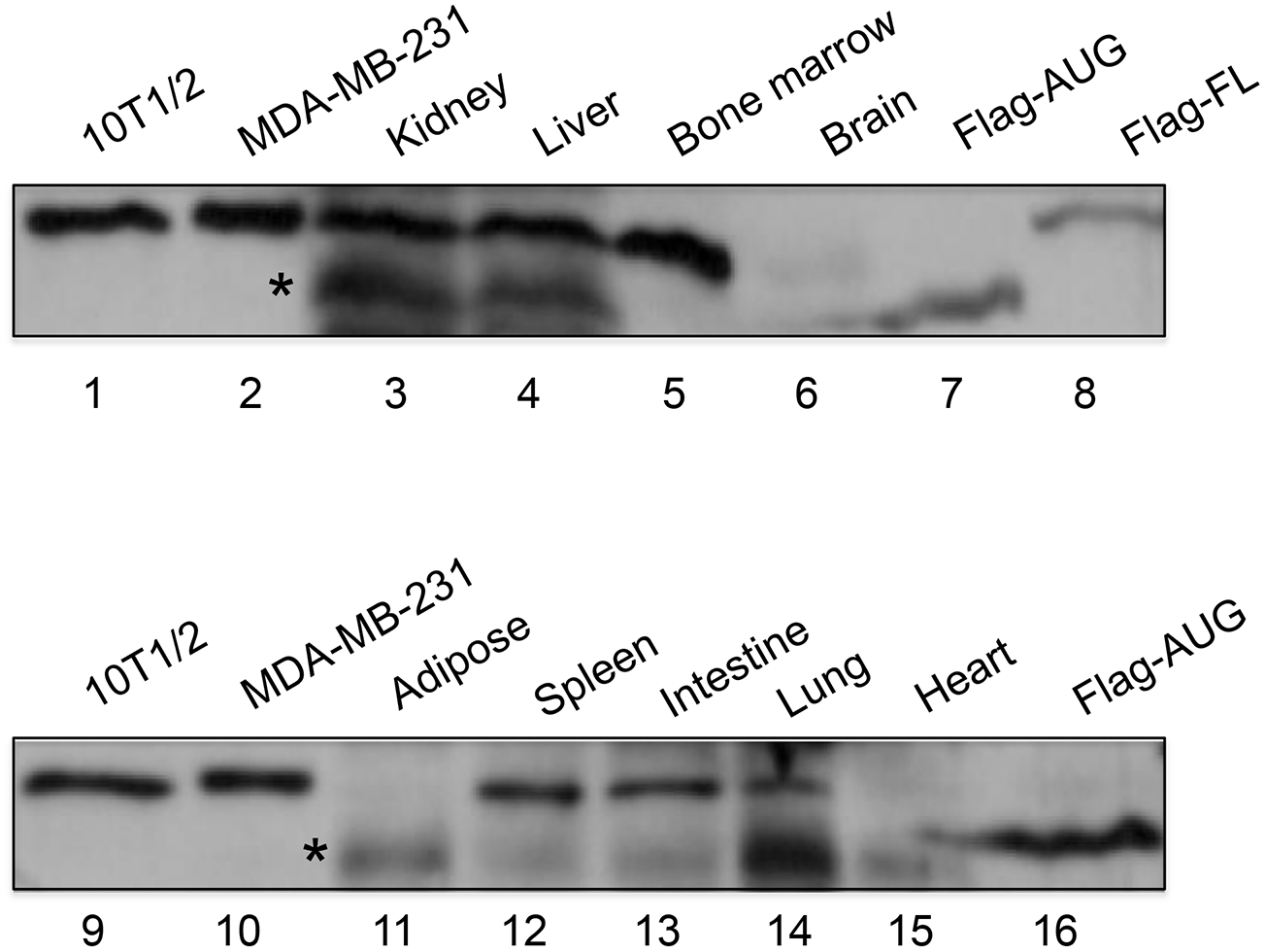
Expression of NELF-B in mouse tissues. Western blots of endogenous NELF-B protein in different mouse adult tissues Aterisks indicate the shorter version of the endogenous protein. 10T1/2: lysate from a mouse adipose progenitor cell line; MDA-MB-231: lysate from a human breast cancer cell line; Flag-AUG and Flag-FL: Flag-tagged mouse NELF-B ectopically expressed in MEF depleted of endogenous NELF-B. Fifty and thirteen μg of total tissue lysates were loaded in the top and bottom gels, respectively.

Our current work identifies a non-canonical transcription initiation site for NELF-B. The preference of this newly discovered non-AUG codon over the annotated AUG is most likely due to the sequence context surrounding the initiation codon that is more favored by the translation machinery. Consistent with the *in vitro* findings, the majority of mouse adult tissues surveyed exclusively express the full-length NELF-B protein (Fig 6). However, it is interesting to note that lysates of several tissue sources including kidney, liver, adipose, and lung also contain a short version that migrated at the approximate position as the AUG-initiated protein (Fig 6). Although the exact identity of the shorter version of the endogenous NELF-B protein requires further validation, the survey of mouse tissue strongly suggests that full-length endogenous NELF-B derives from a longer open reading frame than the predicted coding sequence based on the canonical AUG. Our *in vitro* study indicates that the amino acid sequence upstream of the annotated AUG is not critical for the assembly of the NELF complex or NELF-dependent cell growth *in vitro*. However, it remains plausible that alternative translation initiation of mouse NELF-B is regulated to confer tissue-dependent functional needs.

## Materials and Methods

### Cells

11.5-day-old embryos that carried one deleted allele and one floxed allele of *Nelf-b* (*Nelf-b* ^*fl/-*^) were used to isolate MEFs. Animal work was approved by IACUC at the University of Texas Health Science Center at San Antonio. Per recommendation by the Panel on Euthanasia of the American Veterinary Medical Association, animals were euthanized via CO2 inhalation. The mice were sacrificed specifically for the purpose of this study. Cells were immortalized according to the published protocol [44]. Immortalized MEFs were then cultured in high glucose Dulbecco's modification of Eagle's medium (Gibco; #11965–092) supplemented with 10% fetal bovine serum (Gibco; #16000–044) and 1X antibiotics (Gibco; #15140–122). Human embryonic kidney 293T cells (ATCC, CRL-3216) were cultured in DMEM supplemented with 10% FCS, 1X antibiotics, 0.1 mM MEM non-essential amino acids (Gibco; # 11140–050), 1X L-Glutamine (Gibco; # 25030–081) and 1 mM MEM Sodium Pyruvate (Gibco; # 11360–070).

### Plasmid Construction

The template for the predicted coding sequence (CDS) of mouse *Nelf-b* that starts from the annotated AUG was used in a previous study [43]. After PCR amplification, Untagged *Nelf-b* CDS (AUG), N-terminal Flag-tagged CDS (Flag-AUG), and C-terminal Flag-tagged CDS (AUG-Flag) were then subcloned into a retrovirus expression vector pBabe-neo (Addgene, # Plasmid 1767). The template for 5’UTR of mouse *Nelf-b* was amplified from the genomic DNA of C57BL/6J mouse strain. Ligation-PCR was used to generate the full-length cDNA with the complete 5’UTR sequence (FL). Untagged FL, N-terminal Flag-tagged FL-NELF-B (Flag-FL), and C-terminal Flag-tagged FL-NELF-B (FL-Flag) were then subcloned into pBabe-neo. Sequences of the resulting plasmids were verified by sequencing.

### Site-directed Mutagenesis

Point mutation at various potential initiation codons was introduced using QuikChange Site-Directed Mutagenesis Kit (Stratagene; #200518) according to the manufacturer’s instructions.

### Transient Transfection

Transient transfection of *NELF-B* pBabe-neo plasmids into 293T cells was carried out using Lipofectamine 2000 (Invitrogen, #11668–027). Briefly, 4 x 10^6^ 293T cells per 6-cm dish were plated in growth medium free of antibiotics 24 h before transfection. Cells were transfected according to manufacturer’s instruction and collected for the expression analysis 24 h post transfection.

### Retroviral Infection

Production of the pBabe-neo retrovirus and subsequent viral infection was essentially the same as described previously [43]. The detailed protocol is available at the Addgene website. In brief, 293T cells were transfected with an equal amount of the pBabe vector and a helper vector [45]. Forty-eight hours after transfection, supernatant of the transfected 293T cells was harvested and used to infect 1.5 x 10^5^ MEFs in a 6-well plate seeded one day before infection. MEFs were exposed to virus supernatant containing 8 μg/ml Polybrene (EMD Millipore; #TR-1003-G). The 6-well plate was then spun at 1,500 × g for 4 h at 4°C and incubated at 37°C for 4 h before replacing with fresh growth medium. Geneticin (2 μg/ml, Gibco; #10131–035) was used to select infected MEFs.

#### Adenovirus Infection and Cell Growth

Ad5CMVCre-eGFP and Ad5CMVeGFP adenoviruses were purchased from the Gene Transfer Vector Core Facility at the University of Iowa. Cells were infected using 100 multiplicity of infection (MOI) according to the provided protocol. Three days after viral infection, MEFs proliferation was measured by MTS assay described previously [43].

### Co-immunoprecipitation (co-IP)

MEFs expressing AUG-flag and FL-flag without endogenous NELF-B were used for co-IP of mouse NELF proteins. Approximate 5 x 10^6^ cells were used for each reaction and in 550 μl of lysis buffer containing 20 mM Tris-HCl, pH 8.0; 500 mM NaCl; 1% NP-40; 5 mM EDTA. The following protease inhibitors were added before cell lysis: 1 μg/mL leupeptin (Sigma-Aldrich; # L5793), 1 μg/mL aprotinin (Sigma-Aldrich; # A6103), 1 μg/mL pepstatin (Sigma-Aldrich; # P5318), and 1 mM PMSF (Sigma-Aldrich; # 78830). Lysates were passed through a 27G needle to help disrupt the genomic DNA. The lysates were then centrifuged at 12,000 g for 10 min at 4°C. Fifty μl of the supernatant was saved as input control. The rest of the supernatant was immunoprecipitated overnight with 20 μl of 50% slurry of the anti-Flag agarose beads (Sigma-Aldrich; #A2220) in IP. The beads were washed three times with the lysis buffer at 4°C, each lasting for 30 min. The immunoprecipitates were eluted with 50 μl Flag peptide (Sigma-Aldrich; # F3290) at a final concentration of 100 μg/ml according to the manufacture protocol.

### Western Blot and Antibodies

Standard procedure for western blot was describe previously [46]. NELF-A, NELF-B, NELF-C/D, and NELF-E antibodies were described previously [20,46]. Anti-Flag antibody (Sigma-Aldrich; #A8592), rabbit IgG (Vectorlabs; #I-1000) and anti-α-tubulin antibody (Calbiochem; #CP06) were commercially available.

### Microarray

The gene expression profiling and data analysis were described before [43].

### RNA Secondary Structure Prediction

Prediction of secondary RNA structure was performed using the RNAfold web server (http://rna.tbi.univie.ac.at/cgi-bin/RNAfold.cgi). Settings were used to yield the minimal free energy and avoid any isolated base pairs. For mouse NELF-B, a 70 bp-long fragment immediately downstream of CUG-143 was used for the prediction.

## Supporting Information

S1 Table Gene Expressing Profiling and Gene Ontology Using FL and AUG-NELF-B Expressing MEFs. (XLSX)
